# Hybrid SPECT/CT Imaging in the Evaluation of Coronary Stenosis: Role in Diabetic Patients

**DOI:** 10.5402/2013/419737

**Published:** 2012-09-10

**Authors:** Andrea Romagnoli, Orazio Schillaci, Chiara Arganini, Eleonora Gaspari, Aurora Ricci, Daniele Morosetti, Irene Coco, Sonia Crusco, Ferdinando Calabria, Massimiliano Sperandio, Giovanni Simonetti

**Affiliations:** ^1^Dipartimento di Diagnostica per Immagini, Imaging Molecolare, Radiologia Interventistica e Radioterapia, Fondazione Ospedaliera Policlinico “Tor Vergata”, Viale Oxford 81, 00133 Roma, Italy; ^2^Dipartimento di Medicina Nucleare e Neuroradiologia, IRCCS Neuromed, Via Atinense 18, 86077 Pozzilli, IS, Italy

## Abstract

*Purpose*. Our purpose was to combine the results of the MDCT (multidetector computed tomography) morphological data and the SPECT (single-photon emission computed tomography) data using hybrid imaging to overcome the limits of the MDCT in the evaluation of coronary stenosis in diabetic patients with large amount of calcium in the coronary arteries. *Method and Materials*. 120 diabetic patients underwent MDCT examination and SPECT examination. We evaluated 324 coronary arteries. After the examinations, we merged CT and SPECT images. *Results*. CT evaluation: 52 (32.8%) coronaries with stenosis ≥ 50%, 228 (70.4%) with stenosis < 50%, and 44 (13.6%) with a doubtful evaluation. SPECT evaluation: 80 (24.7%) areas with hypoperfusion, 232 (71.6%) with normal perfusion, and 12 (3.7%) with a doubtful evaluation. Of 324 coronary arteries and corresponding areas, the hybrid SPECT/CT evaluation showed 92 (28.4%) areas with hypoperfusion, and 232 (71.6%) with normal perfusion. *Conclusion*. Hybrid CT/SPECT imaging could be useful in the detection of significant coronary stenosis in patients with large amount of coronary calcifications.

## 1. Introduction

Diabetes mellitus type 2 is strictly related to CAD (coronary artery disease); in fact 70–80% of diabetic patients die of cardiovascular complications; moreover, these patients have a risk of myocardial infarction about four times higher than that found in the general population [1–3]. Therefore, the early detection of CAD in diabetic patients is very important.

Several noninvasive techniques are available for this purpose, including stress ECG and SPECT (single photon emission computed tomography) [4, 5] and most recently coronary CT. In fact the latest CT generation with 64 slices has emerged as a truthful alternative to conventional CA (coronary angiography), with excellent diagnostic accuracy allowing us to identify and quantify the degree and extent of coronary artery disease, including the study of wall arteries [6–9]. 

Previously published results have proved the high values of sensitivity, specificity, and negative predictive value (almost 100%) of CT for the assessment of coronary disease, even in patients treated with stent and bypass [10, 11]. However, in case of important coronary calcifications, the CT examination presents several limitations in residual vessel lumen evaluation [12].

Otherwise, MDCT (multidetector computed tomography) sometimes shows some limitations in the grading of coronary stenosis due to motion artefacts or severe vessel calcifications. It is well known that diabetes causes a large amount of vessel calcification, resulting in lower diagnostic accuracy of CT in the detection and evaluation of coronary stenosis in diabetic patients [13]. In this case, it could be useful to work with hybrid imaging, merging the anatomical images of CT to the functional images of SPECT, overcoming the limits of the two techniques.

Our purpose was to combine the results of the MDCT morphological data and the SPECT data using hybrid imaging to overcome the limits of the MDCT in the evaluation of coronary stenosis in diabetic patients with large amount of calcium in the coronary arteries.

## 2. Method and Materials

### 2.1. Population

Between January 2009 and December 2011, we enrolled 120 consecutive diabetic patients (84 males and 36 females), mean age 67 years old (range 50–78), and performed a coronary CT. All the patients had one or more cardiac symptoms such as stable angina, atypical chest pain, and dyspnea. 

We evaluated the cardiovascular risk factors for each patient, looking for hyperglycaemia (glycated haemoglobin > 7.5%), hypertension (BP > 130/85 mmHg), hypercholesterolemia (cholesterol > 190 mg/dL), obesity (BMI ≥ 30 Kg/cm^2^), smoking, and family history of CAD (Table 1). 

Twenty patients with a history of known CAD (1 with previous bypass graft surgery, 19 with previous successful angioplasty) were included, while patients with a serious arrhythmia, known allergy to iodinated contrast agents and kidney failure, were excluded.

The patients underwent a gated-SPECT examination within 5 days.

Invasive coronary angiography was performed in patients in which the CT examination results were doubtful. Afterwards the imaging fusion results were compared to the coronary angiography evaluation.

All patients, provided informed consent to the examinations and the study was approved by the local ethics committee.

### 2.2. CT Scan

We used a 64-slice CT scanner (LightSpeed VCT, General Electric Medical Systems, Milwaukee, WI, USA) and a retrospective synchronization technique. Patients with a heart rate higher than 65 bpm were previously (almost 5 days before) treated with oral beta-blocker therapy.

A preliminary unenhanced scan was done to determine the scan extent and to calculate the calcium score (SmartScore protocol). The acquisition stack extended from the ascending aorta superiorly (approximately 1 cm above the tracheal bifurcation) and the heart apex inferiorly (lower portion of the lowest hemidiaphragm), therefore enabling the evaluation of the entire cardiac volume. Scan parameters for the unenhanced baseline scan were beam collimation 8 × 2.5 mm, slice thickness 2.5 mm, table feed 1 cm/4 slices, tube rotation speed 0.35 s, tube voltage 120 kV, intensity 300 mA, FOV 25 cm, craniocaudal scan direction.

A second image stack was then acquired after intravenous administration of iodinated contrast material using a dual-head automated injector (Stellant, MEDRAD, Pittsburgh, PA, USA). A dose of 80 mL of nonionic iodinated contrast material (Iomeron 400, Bracco, Milan, Italy) was administered through an 18-Gauge needle cannula placed in an antecubital vein, followed by 40 mL of saline solution, both at a rate of 5 mL/s. To synchronize the beginning of the scan with the arrival of the contrast agent in the coronary arteries, the bolus-tracking technique was used. Parameters for the contrast-enhanced scan were beam collimation 64 × 0.625 mm, slice thickness 0.625 mm, reconstruction increment 0.625 mm, table feed 2.9 mm/rotation, tube rotation 0.35 s, tube voltage 120 kV, intensity 400–800 mA, FOV 25 cm, craniocaudal scan direction. Scan duration was 5.5 s (range 4.2–6.8 s). Image reconstruction was carried out using three temporal windows at 70%, 75%, and 80% of the cardiac cycle, corresponding to the *R*-*R* interval or mid to end diastole. In the event of motion artefacts due to sudden changes in heart rate, other reconstruction windows were used (from 40% to 65% of the *R*-*R* cycle). The mean radiation exposure dose for patient population was 16.3 mSv.

### 2.3. CT Image Analysis

The CT datasets were analysed by the agreement of two independent, experienced readers, who used axial source images, multiplanar reformations, volume rendering, and thin-slab maximum-intensity projections on a remote workstation (Advantage Workstation 4.4; GE Healthcare). Each coronary was judged as negative for coronary disease in the presence of one stenosis < 50% and positive in the presence of one stenosis ≥ 50% [14–16].

### 2.4. SPECT Scan

Gated-SPECT was performed on a double-headed camera system (SPECT-TC VG Millennium, GE Healthcare, USA).

We adopted the single day protocol: stress-rest protocol with 300 MBq ^99m^Tc-MIBI or tetrofosmin at peak exercise during bicycle ergometry or dipyridamole infusion and 900 MBq at rest (*n* 203, 77.2%) after 3 hours. SPECT images were acquired 1 hour after the radiopharmaceutical injection both for the rest and the stress phases.

Thirty-two images of 25 sec per frame (matrix 64 × 64, zoom 1.33) were acquired using the “step and shoot” technique (90 g/head). Energy discrimination was provided by a 20% window centred over the 140 KeV photon peak of ^99m^Tc. 

### 2.5. SPECT Image Analysis

Transverse images were reconstructed by the filtered back projection method, with a Butterworth filter (order 10; cutoff 4.0) for processing and a Ramp filter for back projection using a Xeleris console.

Scintigraphic images for stress and rest were evaluated semiquantitatively by an experienced observer. The left ventricular myocardium was divided up into 17 segments; each of the 17 segments was scored according to the guideline for semiquantitative analysis (Semiquantitative Scoring System: the five point model: 0 = normal; 1 = mildly reduced-not definitely abnormal; 2 = moderate reduced-definitely abnormal; 3 = severe reduced; 4 = absent radiotracer distribution) [17]. 

### 2.6. Hybrid SPECT/CT Images

The fusion between stress-SPECT images and CT axial images was done using the Cardiac IQ Fusion protocol on a remote workstation (Advantage Workstation 4.4; GE Healthcare). This software allows a perfect matching between the images using as reference points the CT myocardium outlines and the SPECT epicardial surface of the left ventricle; furthermore this software cuts the aorta and the veins. Several 3D protocols permit a better visualization of the coronaries, the wall septum, and the right ventricle. The result of the fusion is an image with a high spatial resolution and a better definition of the coronary stenosis.

These hybrid images have been evaluated separately by a radiologist and a nuclear medicine doctor with eventual agreement.

### 2.7. Conventional Coronary Angiography

CCA was performed using a Philips flat-panel system with multiple projections (Medical Philips System, The Netherlands). The video clips were analysed by a blind observer using quantitative software. Stenoses were evaluated as a percentage of the reference diameter determined in two orthogonal projections, taking the mean of the two samples as the final value. A value ≥50% was considered a significant stenosis.

### 2.8. Statistical Analysis

All data were entered into a database for statistical processing. Data were expressed as means plus one standard deviation (SD) or as percentages. Diagnostic accuracy for evaluating significant stenoses was calculated by comparing SPECT/TC with CCA images. The comparison between groups was obtained by analysis of variance or the *χ* ^2^ test, as appropriate. Statistical significance was set at *P* < 0.05.

## 3. Results 

### 3.1. CT Results

CT study was performed without complications in all patients. Mean heart rate of the patients during the examination was 65 bpm (range 56–77 bpm).

After the CT scans, 12 (10%) patients were excluded because of motion artefacts due to an increase in heart rate during the acquisition.

The quality of the images of the other 108 (90%) patients was excellent in 76 patients (70.2%), good in 16 patients (15%), sufficient in 8 patients (7.4%), and low in 8 patients (7.4%). The mean *calcium score* value was 453 ± 21 (AGATSTON SCORE). 

Considering the three main coronary branches in the overall count, left anterior descending (LAD) artery, right coronary artery (RCA), and left circumflex artery (LCX), 324 coronaries were evaluated.

A total of 52 vessels (32.8%) had stenoses >50%, in particular 28 (8.6%) were in the LAD, 16 (4.9%) in the RCA, and 8 (2.4%) in LCX; 228 coronaries (70.4%) were found to have nonsignificant stenoses (<50%) (Table 2). We observed that 44 coronaries (13.6%) were not evaluable or doubtful due to the presence of a large amount of vessel calcification (Table 2). The mean calcium score value in not evaluable or doubtful coronaries was 823 ± 36.

### 3.2. SPECT Results

Considering the 324 areas corresponding to the CT coronary arteries, we detected 80 (24.7%) perfusion defects (Table 2). In particular, 44 (13.6%) perfusion defects were located in the anterior and septal wall, 28 (8.6%) in the inferior wall, and 8 (2.5%) in the lateral wall.

Furthermore, gated-SPECT showed 232 (71.6%) areas without perfusion defects and 12 (3.7%) doubtful cases because of artefacts due to mammary gland attenuation in the female patients (Table 2).

### 3.3. Comparison of CT versus SPECT

The 52 (16%) coronary arteries with significant stenosis at CT were confirmed as perfusion defects at SPECT.

Of 228 coronaries (70.4%) found to have nonsignificant stenosis at CT, 212 (65.4%) were confirmed as normal perfusion areas while 16 (4.9%) were described as perfusion defects at SPECT.

Of 44 (13.6%) nonevaluable coronaries at CT, 12 (3.7%) were described as perfusion defects at SPECT, 20 (6.2%) as normal perfusion areas, and 12 (3.7%) as doubtful cases because of artefacts due to mammary gland attenuation in the female patients (Table 3).

### 3.4. Hybrid SPECT/CT Results and Comparison versus CT

Of 324 coronary arteries and corresponding areas, the hybrid SPECT/CT evaluation showed 92 (28.4%) of areas with hypoperfusion and 232 (71.6%) with normal perfusion (Table 2). 

**Figure 1:**
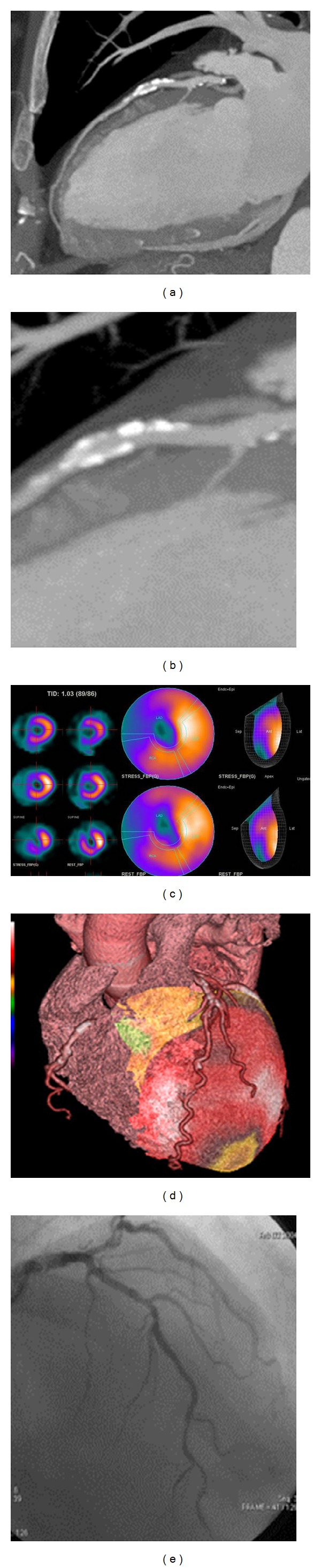
MPR reconstruction anterior descending artery and detail of multiple calcified in the proximal and middle parts ((a), (b)). SPECT images acquired after stress test and rest test showed a hypoperfusion of apex anterior-septal wall of left ventricle (c). Image fusion of the same case shows hypoperfusion of the area served by anterior descending artery (apex; anterior-septal wall) (d). Coronary angiography showed the presence of a stenosis at the middle third of the anterior descending artery (e).

In the first group of 52 (16%) coronaries with significant stenosis at CT and perfusion defect at SPECT, hybrid images demonstrated a hypoperfusion (Figure 1).

**Figure 2:**
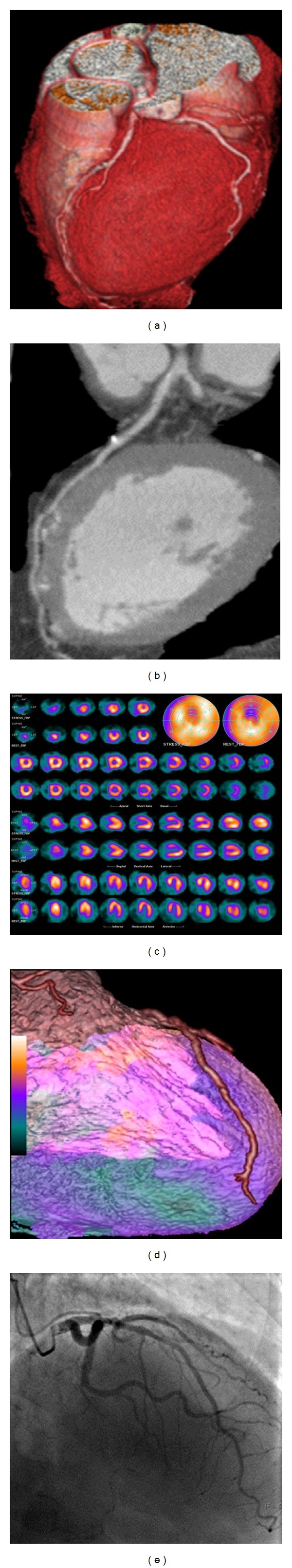
CT volume rendering and curved ((a), (b)) of the anterior descending coronary artery did not show any stenotic lesions. SPECT images, in stress and rest acquisitions, and image fusion showed reduced uptake in the basal lower wall apex of left ventricle ((c), (d)). Coronary angiography showed no focal lesion of the coronary arteries (e). Thus, even if an hypoperfusion was present, it was caused by an artifact.

In the second group of 228 (70.4%) with nonsignificant stenosis at CT, hybrid images demonstrated a normal perfusion in 212 (65.4%) cases and a low hypoperfusion in 16 (4.9%) cases (Figure 2); of these 16 cases, 12 (3.7%) cases were described at CT as stenosis of 40–50%, while the other 4 cases were seen at CT as nontransmural myocardial necrosis (with known history of non-ST infarction).

In the third group of 44 (13.6%) doubtful coronaries at CT, hybrid images demonstrated 20 (6.2%) normal perfusion areas and 24 (7.4%) hypoperfusion areas (seen at SPECT as 12 perfusion defects and 12 doubtful cases), described as nonquantifiable calcified stenosis at CT and were finally evaluated as significant stenosis (Table 3).

The third group of 44 coronaries was also studied with a conventional CA, confirming the results of hybrid images (24 significant stenoses and 20 nonsignificant stenoses). The patients with 24 significant stenoses also underwent a PTCA (percutaneous transluminal coronary angioplasty) with stenting. Therefore the evaluation of hybrid images permitted the identification of all the stenoses and solved the doubts related to the 44 coronaries not well evaluated at CT and SPECT separately, with a diagnostic accuracy of 100% in comparison versus CA (Table 3).

## 4. Discussion

A complete evaluation of coronary artery disease requires both functional and anatomical studies. Myocardial perfusion scintigraphy represents the most widely used technique validated in the prognostic stratification of diabetic patients with known or suspected ischemic heart disease in order to predict the short and medium terms (generally 1-2 years) for cardiac events such death and myocardial infarction [18, 19]. 

However, the perfusion images obtained with SPECT show a good sensitivity and a low specificity in the detection of patients with CAD. 

The diagnostic accuracy of myocardial scintigraphy is affected by different variables.

Unlike positron emission tomography (PET), single-photon emission computed tomography (SPECT) provides no correction for attenuation.

This causes the presence of false positives in some areas (false hypoperfusion obtained from attenuation of photons through the tissues of the body), along the passage from the heart to the gamma camera.

Mostly the artefacts are related to the hepatic uptake, due to the interposition of the diaphragm and, in female patients, in the anterior-lateral wall due to the mammary gland; because of this reason, in our study, SPECT was unable to assess the myocardial perfusion in 12 female patients.

Furthermore SPECT reported some false positives when a patient cannot reach 85% of the theoretical maximum heart rate during the exercise testing; this event occurs in obese patients, in older patients, in patients with arterial disease of the lower limbs, and in subjects treated with beta-blockers. In these cases it would be better to use the dipyridamole testing instead of the exercise testing.

Because of these reasons, there is a high variability of values of sensitivity and specificity reported in previously published meta-analysis [18–21].

Specificity and sensitivity have been improved by the introduction of gated technique: especially in women, the number of false positives can be reduced, observing at the same time the perfusion and the functionality of the myocardial region.

However, sometimes the myocardial region that seems to have a normal perfusion after a stress test could hide a perfusion defect and would not be able to determine a myocardial stunning [21, 22]. 

Coronary CT, when used in selected populations of patients with low-intermediate cardiovascular risk, has been shown to provide essential morphological information about the coronary tree.

The diagnostic accuracy of cardiac CT is widely demonstrated by high values of sensitivity, specificity, and negative predictive value [23, 24].

Actually, the most important limit in the evaluation of the vessel lumen is the presence of large coronary artery calcification, causing a blooming type artefact, due to the hardening of the X-ray beam during the passage through the calcified plaques [25]. This artefact could cause a significant invalidation of lumen visualization determining false positive in coronary CT and consequently sending a certain number of patients with stenosis < 50% to undergo a CA.

In our study the patients, being diabetic, had large calcifications of the coronary arteries, creating doubts in the interpretation of quantitative coronary stenosis in 44 coronary arteries.

During the postprocessing phase, the use of some convolution filters (kernel) can decrease the blooming artefact; in particular applying the high-resolution filters (Bone and Detail), the relative density of calcium is reduced, the margins of calcification plaque are better delineated, and the width of the vascular lumen increases.

However, in a large number of cases, the quantification of stenosis caused by calcified plaque remains still difficult and it is often necessary to send the patient to CA examination or a SPECT. Part of medical literature has tried to define the AGATSTON values above which performing CT would be inappropriate. However, although sensitivity and specificity are reduced, the overall diagnostic accuracy is not reduced and, in our study, it has been implemented with the fusion imaging CT/SPECT [12].

The skillful use of hybrid images, obtained by merging CT and SPECT images, is to have both morphological and functional data with a high spatial resolution, allowing a better stratification of diabetic patients, identifying the patient with a significant stenosis and hypoperfusion who needs revascularization and avoiding a CA in patients with unquantifiable calcification plaques at CT and SPECT [26, 27].

The hybrid images can overcome the limits of coronary CT, permitting the evaluation of large vessel calcification, metal stents, and small size vessels.

However, this problem usually does not occur in the evaluation of noncalcified native coronary arteries; in fact Ong et al. (2006) demonstrated that in patients with calcium score <142 Agatston score the interpretability of the coronary tree was 93.6% against 86.9% of those patients with values >142 Agatston score [28].

In our experience, hybrid SPECT/CT images provide help in the evaluation and grading of calcified stenoses in CT and overcome the problem of the abnormal uptake of the radiopharmaceutical by the breast tissue in SPECT.

In particular in the group of 44 (13.6%) doubtful coronaries at CT, hybrid SPECT/CT images demonstrated 20 (6.2%) normal perfusion areas and 24 (7.4%) hypoperfusion areas (seen at SPECT as 12 perfusion defects and 12 doubtful cases because of breast tissue), described as unquantifiable calcified stenosis at CT and finally evaluated as significant stenosis.

Therefore the evaluation of hybrid images permitted the identification of all the stenoses and solved the doubts related to the 44 coronaries not well evaluated at CT and SPECT separately, with a diagnostic accuracy of 100% in comparison to CA.

These results are extremely interesting when applied to a group of patients such as diabetics, in which, until now, the limit of the coronary CT examination in the evaluation of calcified coronary was high [24].

On the other hand, the correct identification of an area of myocardial hypoperfusion at SPECT examination is enhanced by the morphological information of the coronary arteries with a significant decrease in the number of false positive and an increase of the prognostic value of scintigraphy.

In fact, SPECT provides a high prognostic accuracy in the prediction of cardiac event (especially in the short-term followup), although it gives only functional information. The perfusion defects can be localized and assigned to a specific coronary artery only if the SPECT exam is complemented by a morphological assessment.

According to medical literature, hybrid imaging adds diagnostic clinical value, improving patient risk stratification and showing a higher sensitivity and specificity in the detection of the haemodynamically relevant coronary artery stenoses compared to the side-by-side analysis [29–31]. Furthermore the improvement of diagnostic accuracy was obtained with the latest CT scanners, as high-pitch dual-source CT coronary angiography and 320-row cardiac CT, which allow to improve the imaging of coronary plaque and obtain the evaluation of myocardial viability with a high diagnostic accuracy, a reduced radiation exposure and amount of contrast agent, even in patients with heart rates of >80 bpm or atrial fibrillation [32–35].

Our study has the limit of a high exposure of radiation because the patients underwent two different examinations (CT and SPECT) and because we used a retrospective gating during the CT scan; nevertheless, the actual use of the prospective gating and the application of new advanced reconstruction techniques that reduce image noise and improve low contrast detectability and image quality can give higher diagnostic performance of CT scan at very significant lower dose [36, 37]. Another limitation of the present study was the only anatomical and not functional evaluation of coronary stenoses.

Therefore, the usefulness of the fusion between CT and SPECT images is to overcome the limits of CT and the limits of SPECT with a high diagnostic accuracy, especially in patients with high cardiovascular risk and high presence of physical limitations, like the diabetic patients.

## Figures and Tables

**Table 1:** Clinical characteristics of the patient population.

Age (range years)	56–77
Men (no.; %)	84; 70%
Women (no.; %)	36; 30%
Cardiovascular risk factors	
Hypertension (no.; %)	80; 66.7%
Hypercholesterolemia (no.; %)	76; 63.3%
Diabetes mellitus (no.; %)	120; 100%
Obesity (BMI ≥ 30 Kg/cm^2^) (no.; %)	8; 6.7%
Current smoking (no.; %)	44; 36.7%
Family history of CAD (no.; %)	32; 26.7%
Medical history	
Previous myocardial infarction	20; 16.6%
Previous CABG	4; 3.3%
Previous PTCA	16; 13.3%
Dyspnea	31; 13.3%
Typical/atypical chest pain	42; 35%

BMI: body mass index; PTCA: percutaneous transluminal coronary angioplasty; CABG: coronary artery bypass grafting.

**Table 2:** Comparative analysis of CT, SPECT, and CT/SPECT data.

CT	SPECT			CT/SPECT		
	Perfusion defect (no.; %)	No hypoperfusion (no.; %)	Doubts (no.; %)	Perfusion defect (no.; %)	No hypoperfusion (no.; %)	Doubts (no.; %)
Stenosis ≥ 50% (no.; %) 52 (16.1%)	52 (16%)	—	—	52 (16%)	—	—
Stenosis < 50% (no.; %) 228 (70.3%)	16 (4.9%)	212 (65.4%)	—	16 (4.9%)	212 (65.4%)	—
Stenosis doubts (no.; %) 44 (13.6%)	12 (3.7%)	20 (6.2%)	12 (3.7%)	24 (7.4%)	20 (6.2%)	—

**Table 3:** Comparative analysis between CT/SPECT and coronary angiography of 44 stenoses with doubt interpretation of CT examination.

	Coronary angiography		CT/SPECT	
	Stenosis ≥ 50% (no.; %)	Stenosis < 50% (no.; %)	Perfusion defect (no.; %)	No hypoperfusion (no.; %)
Stenosis doubts (no.; %) 44 (13.6%)	24 (7.4%)	20 (6.2%)	24 (7.4%)	20 (6.2%)
